# Tongue Compressor

**Published:** 1865-02

**Authors:** 


### TONGUE COMPRESSOR.

For the original idea of this simple but very useful instrument we are indebted to Dr. Geo. E. Hawes of New York City.

The instrument is not in general use—it is used by a few, and by them highly appreciated. It has not till recently been properly constructed; in this perhaps lies the fact that it has not been more generally used.

This is a very efficient instrument for accomplishing the work indicated by the name. It consists of a base or lower portion of heavy plate of about an inch and a third in width, and an inch and two thirds long, concave to accommodate the base of the chin. To the front of this base is attached a square heavy tube, about three lines in diameter and about an inch and a half in length; a spring ratchet is attached to it that passes into the tube and holds the notched bar that plays in it. To this bar the tongue piece is attached which is made forked, and of such width as to pass freely beween the teeth of the two sides of the lower jaw, it should have such form as not to cut or irritate the tongue or gums.

With this instrument the tongue may be clamped down in place and kept in position as long as desired. The sublingual and submaxillary ducts may be very effectualy closed by placing upon them rolls or pads of bibulous or blotting paper before applying the compress, a pad of paper or cloth should also be placed on the tongue before applying the instrument.

In filling the inferior molars and bicuspids we regard this as an indispensable instrument. No one should be without it.

